# Self-assembly mechanism of Ni nanowires prepared with an external magnetic field

**DOI:** 10.3762/bjnano.6.217

**Published:** 2015-11-09

**Authors:** Xiaoyu Li, Hu Wang, Kenan Xie, Qin Long, Xuefei Lai, Li Liao

**Affiliations:** 1 School of Chemical Engineering, Sichuan University, Chengdu 610065, PR China

**Keywords:** chemical reduction, external magnetic field, Ni nanoparticles, Ni nanowires, self-assembly mechanism

## Abstract

Nickel nanowires with a mean diameter of about 95 nm and lengths of up to 26 μm were prepared by a chemical reduction method in aqueous solution under an external magnetic field. The self-assembly mechanism was investigated in detail. The results indicate that the self-assembly process of Ni nanowires consists of three stages: nucleation and growth, ordered alignment and self-assembly, and deposition on the surface and gaps between the nickel particles. The self-assembly phenomenon occurs only when nickel particles grow to a size of about 60 nm in the reaction system. This critical size, which is proposed for the first time, is very important to comprehend the self-assembly mechanism of Ni nanowires prepared with an external magnetic field.

## Introduction

For the past decades, ferromagnetic (e.g., Fe, Co, Ni) nanowires have raised considerable attention due to their application prospects in magnetic, optoelectronic, electronic, sensor and electrochemical devices [1–6]. In particular, Ni nanowires have been the focus of intense research due to their easy preparation compared with iron and cobalt nanowires. There are several reported routes for the preparation of Ni nanowires including template-based electrodeposition [7–10], block copolymer lithography [11], and wet chemical reduction [12]. Among these methods, template-based electrodeposition is the most widely used to prepare Ni nanowires as highly-ordered and size-controlled nanowires can be obtained with this method. However, additional steps such as preparing and removing the templates are required for this method.

Recently, a self-assembly method employing a magnetic field to prepare ferromagnetic nanowires has been extensively studied because of its simplicity and effectiveness. For example, Wang et al. [13–14] synthesized nickel and cobalt nanowires using a gamma irradiation technique under an external magnetic field. Li et al. [15] synthesized nickel chains under a weak magnetic field by hydrazine reduction in ethylene glycol. Smooth Ni nanowires were prepared by Hu et al. [16] under a stronger magnetic field. Soumare et al. [17] synthesized nickel nanowires with a diameter of 250 nm and a length of several microns via a chemical reduction method with an external magnetic field of 1.4 T. Liu et al. [18] prepared Ni nanowires with a diameter of 50 nm via a hydrazine reduction route under external magnetic field assistance.

However, it should be noted that more attention has been paid to the exploration of preparation systems using the magnetic-assisted method for the shape control of the resulting nanowires under an external magnetic field, as opposed to the self-assembly mechanism of this method. The team of Makoto Kawamori [19–21] proposed a possible mechanism of self-assembly speculated from the morphology of nickel and cobalt nanowires. Meng et al. [22] proposed a similar mechanism. However, no related experiments were designed to prove their proposed self-assembly mechanism. Herein, Ni nanowires are synthesized in aqueous solution by a template-free method combined with chemical reduction and the application of an external magnetic field. Based on a previous study [23–24], this paper focuses on the morphology change during the reaction in order to place the self-assembly mechanism into perspective. Surprisingly, nickel nanowires cannot be formed if the diameter of the nickel nanoparticles does not reach a critical size.

## Experimental

All chemicals were of analytical grade without further purification. All reaction solutions were located between two parallel neodymium magnets (60 × 30 mm^2^) separated 150 mm apart. The magnetic intensity inside the reaction solution was approximately 15 mT, which was measured by a magnetometer. In a typical synthesis, 0.10 g of NiSO_4_·6H_2_O and 0.11 g of C_6_H_5_Na_3_O_7_·2H_2_O were dispersed in 60 mL of distilled water in a 100 mL PTFE beaker. After the temperature of the reaction solution reached 80 °C, 0.25 g of NaOH was added to the solution under strong stirring until it was completely dissolved. Then, 2 mL of 80 wt % N_2_H_4_·H_2_O and 50 µL alkaline sodium borohydride solution (3 g of NaBH_4_ and 1 g of NaOH were dissolved in 20 mL of distilled water) were added into the solution with agitation, and the time was recorded immediately and was considered as the starting time of the reaction. The end time of the reaction was defined as when the solution became transparent. Finally, the resulting products were collected, rinsed three times with distilled water and ethanol. In order to investigate the self-assembly mechanism, a series of experiments similar to the above were accomplished, and the only difference was that these reaction liquids were poured into 400 mL of cooled, distilled water after the reaction progressed for 30 s, 60 s, 120 s, 180 s, 200 s, 220 s, 240 s and 300 s. These products were rinsed after being stored for 12 h.

The size and morphology of the products were observed using a field emission scanning electron microscope (FESEM, JHOL, S-450). The composition and crystallographic properties of the products were characterized by X-ray diffraction (XRD, Philips, X'pert) in the range from 10–80° with a scanning rate of 0.02 °/s. Transmission electron microscopy (TEM) images were obtained using a JEOL JEM-100CX TEM.

## Results

**Figure 1:**
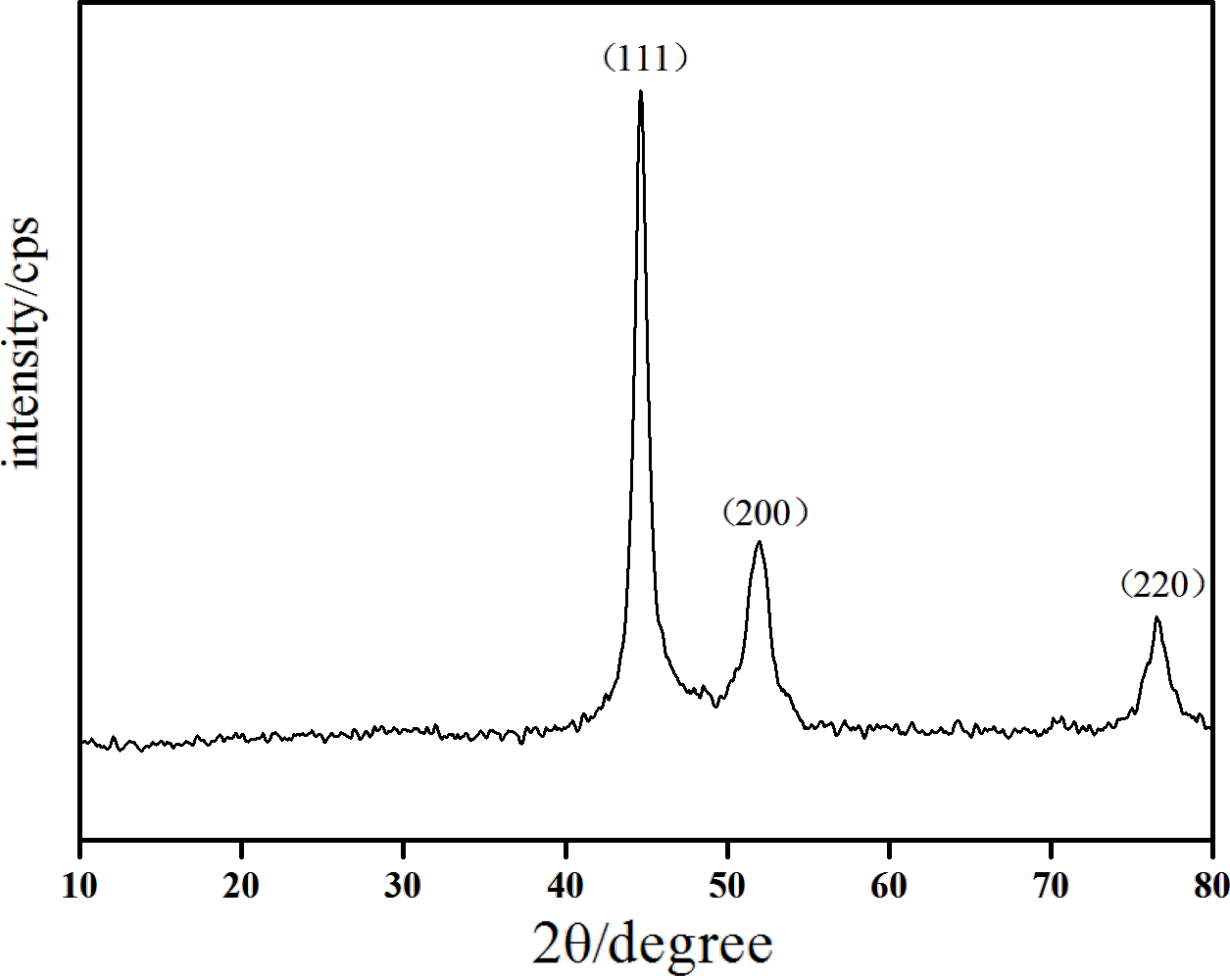
XRD pattern of nickel nanowires.

In Figure 1, the corresponding XRD pattern for the Ni samples is shown. Three characteristic peaks for Ni (2θ = 44.51°, 51.85°, and 76.37°, corresponding to Miller indices (111), (200), and (220)) were found and no impurity peaks were detected. These results indicate that the as-prepared Ni nanowires consisted of pure face-centered-cubic (fcc) Ni (according to powder diffraction file JCPDS 01-1260). It is generally known that nickel is easily oxidized, but peaks of NiO were not observed due to the additional N_2_ produced during the reaction, which protects the Ni nanowires from oxidization. The crystallite size of the Ni nanowires (16 nm) calculated from the (111) fcc Ni peaks by Scherrer’s equation is smaller than the apparent wire diameter (≈95 nm). This indicates that the as-prepared Ni nanowires are polycrystals.

**Figure 2:**
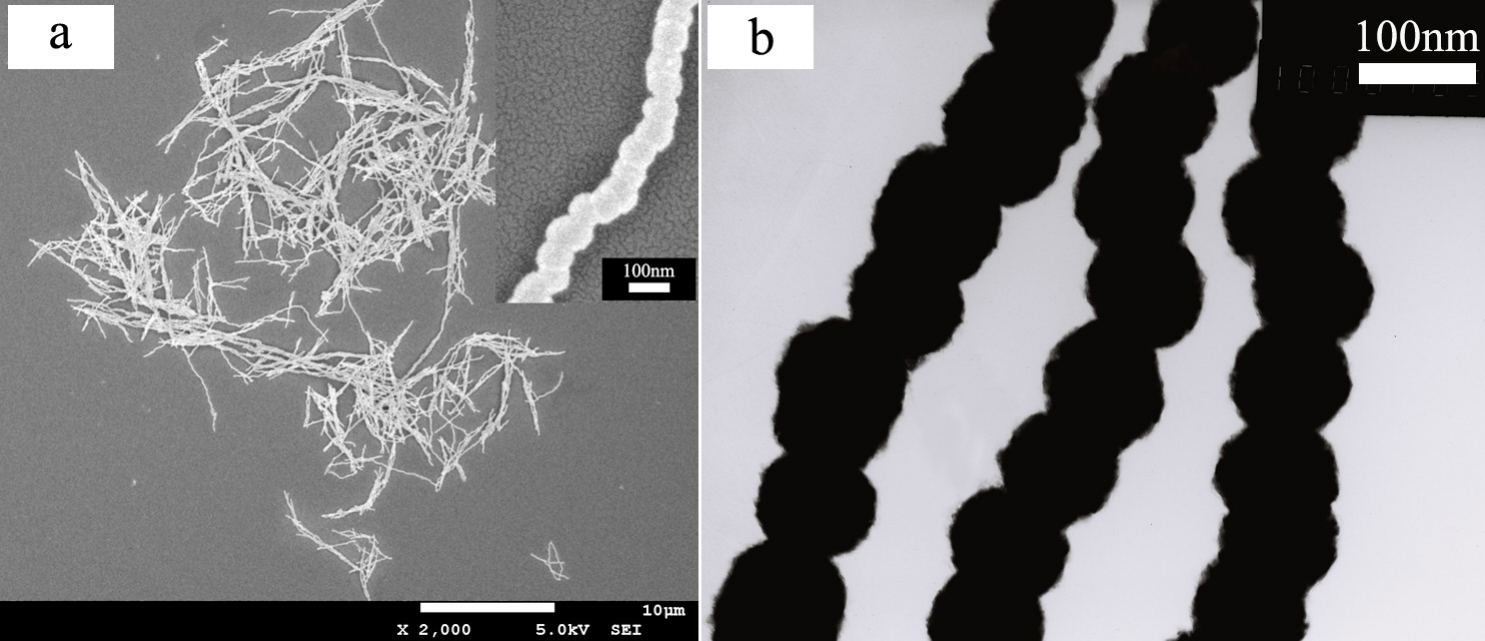
SEM (a) and TEM (b) images of the final Ni samples.

The SEM and TEM images displaying the morphology of the final Ni samples are shown in Figure 2, which indicate that the Ni samples exhibit a chain-like configuration. This implies that the as-prepared Ni nanowires with a diameter of about 95 nm and a length of about 26 μm are composed of spherical-like particles that are connected with each other and form straight chains. Notably, the Ni samples remain chain-like after ultrasonication for 30 min, which implies that the Ni particles grow together and do not simply aggregate. Moreover, the Ni samples likely have a polydomain structure because the single-domain diameter for Ni is about 55 nm with a spherical particle shape [25].

**Figure 3:**
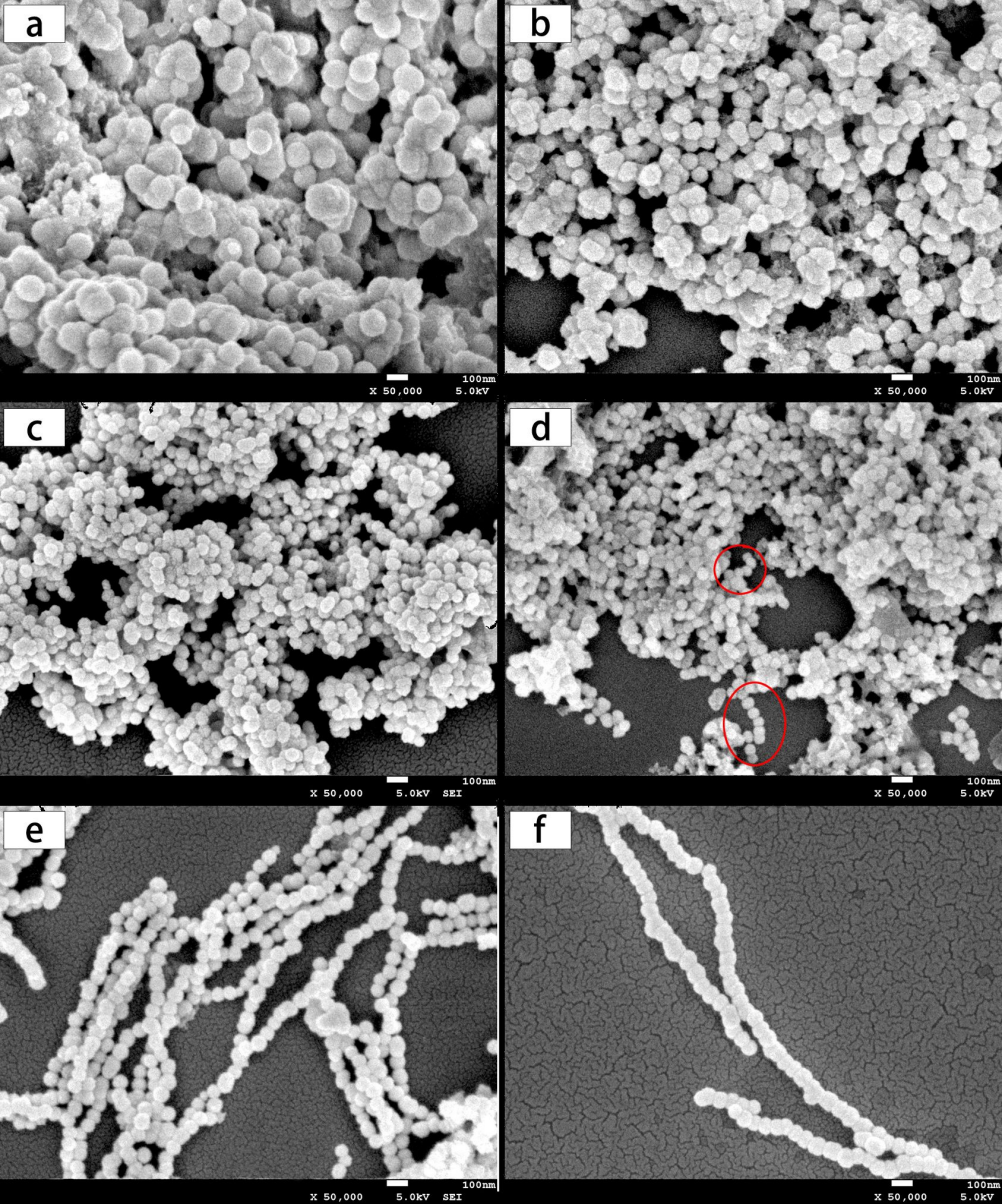
SEM micrographs of products prepared after the reaction proceeded for 30 s (a), 60 s (b), 120 s (c), 240 s (d), 300 s (e), and 600 s (f).

**Figure 4:**
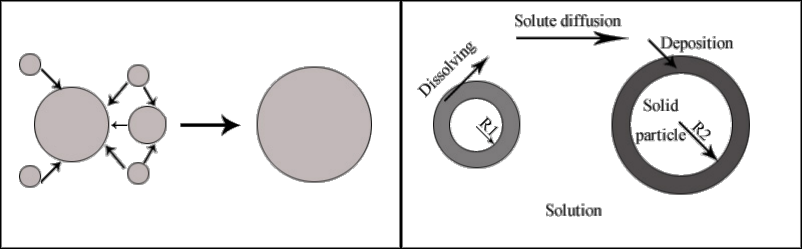
Ostwald ripening: (a) the process of Ostwald ripening involves small particles being engulfed by larger particles; (b) process diagram of Ostwald ripening.

Figure 3 shows the morphology change of Ni nanowires during the reaction. Obviously, only spherical particles can be seen at 30 s (Figure 3a), 60 s (Figure 3b), and 120 s (Figure 3c), while chain-like wires can be seen at 240 s (Figure 3d) although a number of individual particles can also been seen in this graph. Only chains of particles are observed at 300 s (Figure 3e) and 600 s (Figure 3f). It can be inferred that as-prepared Ni nanowires are comprised of connected, single nickel nanoparticles. It is worth noting that the self-assembly phenomenon arises only when nickel particles have grown to a critical size of about 60 nm in the reaction system. Interestingly, the particle diameters in Figure 3a (about 60–125 nm) and Figure 3b (about 70–95 nm) are larger than those in Figure 3c (about 40–55 nm) and Figure 3d (about 55–65 nm). It is known that the total interfacial area of a system must decrease with the time to reach thermodynamic equilibrium by a diffusional mass transfer process from high interfacial curvature regions to low interfacial curvature regions. The interfacial area reduction process is generally called coarsening or Ostwald ripening, which is briefly illustrated in Figure 4 [26–29]. Therefore, this interesting phenomenon may be attributed to Ostwald ripening: while the size of particles is uniformly distributed at 120 s, the difference in size of particles at 30 s and 60 s is larger thus smaller particles dissolve and transfer their mass to the larger particles. The difference in the size of particles at 60 s is smaller than that at 30 s.

## Discussion

In the present system, the product produced by the reduction reaction of NaBH_4_ was induced into nucleation and formed insoluble crystals in the reaction solution. The overall nickel reduction by hydrazine in aqueous solution can be described as follows:

[1]
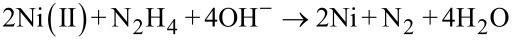


where Ni(II) represents all Ni(II) species in the solution such as Ni^2+^, nickel–citrate complexes, Ni(OH)_2_, [Ni(N_2_H_4_)_3_]^2+^, [Ni(N_2_H_4_)_2_]^2+^ and [Ni(NH_3_)_6_]^2+^ [30]. The above reaction can be separated into an anode reaction (N_2_H_4_ oxidation) and a cathode reaction (Ni deposition).

**Figure 5:**
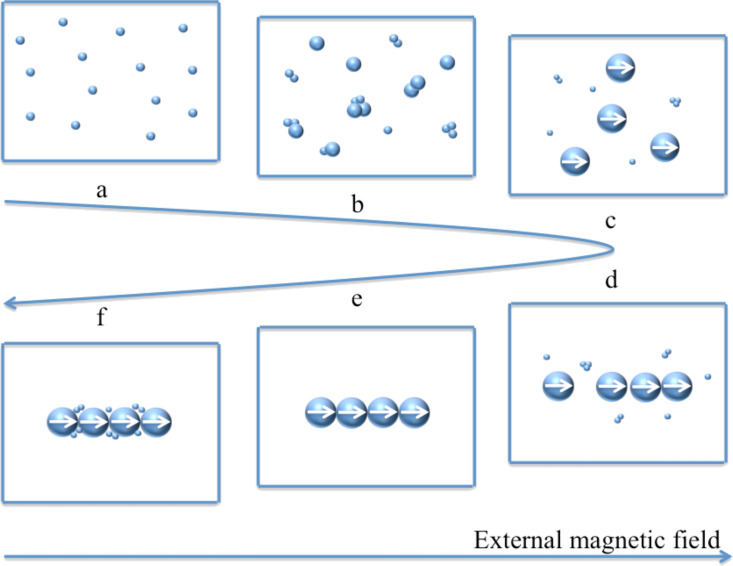
Schematic diagram of the stepwise formation of Ni nanowires.

The possible self-assembly mechanism shown in Figure 5 was deduced from the morphology change of Ni nanowires during the reaction and can be divided into three stages. Firstly, small Ni nanoparticles are generated in the reaction solution by the initiation reaction of NaBH_4_, regardless of the presence of the external magnetic field. Then, these nanoparticles become larger and larger as the reduction reaction of N_2_H_4_ proceeds. These steps, corresponding to Figure 5a and Figure 5b, respectively, can be called the first stage. Nanoparticles are mainly affected by Brownian motion and electrostatic repulsive force in this stage because the magnetic interaction between nanoparticles is too weak to overcome the molecular resistance of the solvent since the dipole magnetic moment of a nanoparticle is proportional to its volume [31]. Similarly, the interaction between the nanoparticle and the external magnetic field is also negligible, thus the motion of Ni nanoparticles cannot be influenced by the external magnetic field. As the reaction proceeds, the diameter of the Ni nanoparticles reaches a critical size where the dipole magnetic moments of the nanoparticles are aligned with the external magnetic field direction, as shown in Figure 5c. Meanwhile, Ni nanoparticles are aligned along the magnetic induction lines (Figure 5d). A nickel particle chain (Figure 5e) is formed since the magnetic interaction between nanoparticles becomes stronger as the size of Ni nanoparticles increases, which results in an enhancement of the van der Walls forces between Ni particles. That is the second stage of the self-assembly mechanism. The critical Ni nanoparticle size has an especially important meaning since it is the inflection point between Ni nanoparticles and Ni nanowires in the reaction system and is the key to the mechanism. It can be defined as the critical size of self-assembly. This is an inevitable consequence of various interaction forces such as magnetic interaction between magnetic dipoles, van der Walls forces, the molecular resistance of the solvent, Brownian motion and electrostatic repulsive forces. Therefore, the critical size of self-assembly is closely related to the magnetic field strength and temperature of the reaction system. In the third phase, Ni(II) preferentially deposits in the gaps between particles to decrease the interfacial energy, as shown in Figure 5f, which can be demonstrated by Figure 3e and Figure 3f. Comparing the diameter of the nanowires in Figure 3e and Figure 3f, it is doubtless that Ni(II) deposits on the surface of the nickel nanowires as well.

Interestingly, magnetic nanoparticles can be first prepared and then aligned into one-dimensional chain-like structures in ferrofluids under a magnetic field [32–35]. The self-assembly behavior is similar to the second stage of the above self-assembly mechanism. Only chain-like nanowires can be prepared by this assembly method, while the above mechanism indicates that Ni nanoparticles, Ni nanowires with chain-like structure and Ni nanowires with smooth surfaces can all be prepared in the given reaction system as long as the reaction is terminated at different stages by cooling with a large amount of distilled water or liquid nitrogen. Of course, the reaction can conclude at different stages by adjusting the process conditions if the critical size of self-assembly is clear for the reaction system. This demonstrates that the critical size of self-assembly is of great significance in order to understand the self-assembly mechanism of Ni nanowires prepared under an external magnetic field.

## Conclusion

Under an external magnetic field, polycrystalline nickel nanowires were synthesized by a chemical reduction method. Further studies indicated that the self-assembly process involves three stages: nucleation and growth, ordered alignment and self-assembly, and the deposition on the surface and in the gaps between nickel particles. Particularly, this self-assembly phenomenon arises only when the diameter of the spherical nickel nanoparticle reaches a critical size, which is the key of the self-assembly mechanism of nickel nanowires. This critical size is also of great importance for preparing nickel nanomaterials (nanoparticles, nanochains and nanowires with a smooth surface) in an aqueous solution system under an external magnetic field.
